# Biotechnology executive order opens door for regulatory reform and social acceptance of genetically engineered microbes in agriculture

**DOI:** 10.1080/21645698.2024.2381294

**Published:** 2024-07-27

**Authors:** Jabeen Ahmad, Amy Grunden, Jennifer Kuzma

**Affiliations:** a Department of Plant and Microbial Biology, North Carolina State University, Raleigh, NC, USA; b Genetic Engineering and Society Center, North Carolina State University, Raleigh, NC, USA; c School of Public and International Affairs, North Carolina State University, Raleigh, NC, USA

**Keywords:** agriculture, biotechnology, CHIPS and Science Act, Coordinated Framework for Regulation of Biotechnology, genetically engineered microbes, regulatory review, 2022 Executive Order

## Abstract

In the United States, regulatory review of genetically engineered microbes for agriculture falls under the Coordinated Framework for the Regulation of Biotechnology (CFRB). However, the lack of a centralized regulatory pathway and multiple oversight authorities can lead to uncertainty in regulatory review. Using three microbial-based technologies for agriculture as illustrative examples, this commentary identifies the weaknesses and challenges associated with the CFRB by assessing the current system and proposed changes to the system under a multi criteria decision analysis framework. In addition, it discusses opportunities for regulatory reform to improve clarity, efficiency, and public acceptance of genetically engineered microbes in agriculture under the CHIPS and Science Act and the 2022 Executive Order on the Bioeconomy.

## Introduction

The Green Revolution significantly improved crop yields and changed agricultural practices globally. The development of high-yielding dwarf cereals, chemical fertilizers and pesticides, and mechanical cultivation prevented hunger for millions of people.^1,2^ With increasing global population growth, climate change, and adverse impacts of chemical agricultural inputs, efforts for the next Green Revolution have focused on sustainable agricultural practices and synthetic biology for food security, crop resilience, and soil health.^3–6^ Agricultural biotechnology has expanded from genetic engineering of crops^3^ to genetic engineering of microbes and microbiomes that support plant growth, development, and resilience.^5^

Plant growth promoting microbes (PGPMs) are bacteria, fungi, and archaea that associate or interact with plants, enhancing their growth and development while helping them survive and thrive under stressful or adverse conditions.^7–9^ PGPMs play an essential role in nutrient acquisition and uptake as well as immunity and defense signaling.^7,8^ While PGPMs perform these tasks naturally, genetic engineering techniques, such as CRISPR editing (Clustered Regularly Interspaced Short Palindromic Repeats), are used to improve their plant beneficial attributes or to add new ones.^10,11^ The genetic alteration of microbes is not new. Genetically engineered microbes have been used for bioremediation, food production, and medicine.^12–18^ Genetic engineering has also been a part of agriculture, with crops modified for yield, stress tolerance, pathogen or pest resistance, and nutritional content.^19–21^

Though genetic engineering has a history of application in agriculture and microbes, genetically engineered microbes for agriculture have not taken off commercially until recently^22^ and present a unique regulatory case as these microbes impact food crops and other plants. They are not food product ingredients like live cultures. Furthermore, while they are not intended to be consumed as part of the edible crop, it is possible that they would be. Genetically engineered microbes also pave the way for genetically engineered microbiomes whereby an entire community or group of microbes are altered.^23^ As new agricultural biotechnology is being developed, it is important to understand the regulatory pathways that the technology will take and assess whether the current regulatory system can effectively and efficiently balance interests between technological development, commercialization, safety, and risk. Using three microbial-based products: PROVEN® 40 product line from Pivot Bio,^22,24^ Poncho® VOTiVO® from BASF,^25,26^ and BiomElixOne® from Folium Science^27,28^ (Table 1) as illustrative examples and multi criteria decision analysis as an assessment tool,^29–31^ we explore the strengths and weaknesses of the U.S. regulatory system in the context of genetically engineered microbes for agriculture and identify gaps and opportunities for improvement in regulation. In addition, we include discussion of the proposed changes to the regulatory system as described by the CHIPS and Science Act and the 2022 Executive Order on the Bioeconomy^32,33^ and discuss whether the proposed changes in the regulatory system address the gaps or weaknesses identified by multi criteria decision analysis as applied to the product examples. Ultimately, we believe that the CHIPS and Science Act and the 2022 Executive Order provide an opportunity to not only improve clarity and efficiency of the U.S. regulatory process but also to increase public approval of genetically engineered technologies through the inclusion of values and other social acceptance factors.

**Table 1.** Three case study microbial-based technologies are explored in this commentary: PROVEN® 40 from Pivot Bio, Poncho® VOTiVO® from BASF and BiomElixOne® from Folium Science. The table summarizes differences between the technologies based on characteristics and regulatory review. The case studies illustrate that modification techniques do not play as significant a role in determining regulatory review pathways. Use and purpose are more relevant criteria.

Case Study Microbial-Based Technologies for Agriculture			
Product Name	PROVEN® 40	Poncho® VOTiVO®	BiomElixOne®
Company	Pivot Bio	BASF	Folium Science
Product Type	Fertilizer supplement, plant health, and nutrition	Pesticide and plant health	Antibiotic and animal health
Purpose	Increase nitrogen fixation	Protect against nematodes and improve root health	Inhibit salmonella in poultry animals
Microbe(s)	*Kosakonia sacchari*, *Klebsiella variicola*	*Bacillus firmus*	*Escherichia coli*
Technique	Guided microbial remodeling using chemical mutagenesis, directed evolution, and site-specific editing (*e.g*. CRISPR/Cas, Zinc Fingers, TALENs)	Non-engineered	Guided biotics using site-specific editing (CRISPR/Cas) and plasmid engineering
General Modifications	Knock-out mutations, alter or eliminate regulatory sequences, alter gene expression, eliminate protein activity	Non-engineered	Alter gene expression, introduce plasmids with target-specific engineered CRISPR arrays
Application	Seed treatment or in-furrow	Seed treatment	Feed additive
Subject to Regulation	No	Yes (EPA and USDA)	Yes (EPA and FDA)
Key Attributes that Subject Technology to Regulation	N/A	Pesticide, control plant pest	Intrageneric organism, feed additive
Website	https://www.pivotbio.com/product-proven40-corn.	https://agriculture.basf.us/crop-protection/products/seed-treatment/poncho-votivo.html.	https://foliumscience.com/products.

### The three case study microbial-based technologies for agriculture represent products that are gene-edited, unmodified, and plasmid modified.

Collectively, PROVEN® 40, Poncho® VOTiVO®, and BiomElixOne® (Table 1) demonstrate different regulatory pathways that can be taken for microbial-based technologies, including not being subject to regulatory review. Each technology involves a microbe as a key component, though the uses and modes of action are different. Use of these examples is meant to illustrate the diversity of microbial technologies being developed for agricultural applications and the uncertainty that can exist with navigating regulatory review of genetically engineered microbes for agriculture.

### PROVEN® 40 from Pivot Bio

Nitrogen is an essential element for plant growth and development and is required for the formation of chlorophyll, amino acids, and nucleic acids.^34^ Plants obtain nitrogen primarily from chemical fertilizers, decomposition, or nitrogen-fixing microorganisms, as plants are only able to use nitrogen in specific forms, such as ammonia or nitrate.^34,35^ Nitrogen-fixing microorganisms can transform atmospheric nitrogen gas into a usable form for the plant. In agriculture, chemical fertilizers have been a significant source of nitrogen, but their use can have unintended environmental impacts.^24,36^ To address this problem, Pivot Bio, a California-based agricultural biotechnology company, developed PROVEN® 40, a microbial fertilizer supplement for maize that includes genetically modified plant root-associated bacteria.^24,36^ Through a process called guided microbial remodeling, Pivot Bio isolated two free-living bacteria, *Kosakonia sacchari* and *Klebsiella variicola*, from maize roots and mapped out their nitrogen fixation, assimilation, and colonization gene networks to target genes for nitrogenase production, nitrogen fixation, ammonium excretion, and root colonization.^36^ Using CRISPR/Cas9 editing, mutagenesis, and other genetic engineering techniques, Pivot Bio optimized the bacteria’s ability to express nitrogenase, fix nitrogen, excrete ammonium, and enhanced their ability to colonize roots.^36^ These changes are described to not only allow the bacteria to fix greater quantities of nitrogen from the atmosphere but also to continue nitrogen fixation throughout the life cycle of the plant.

### Poncho® VOTiVO® from BASF

Plant pests, such as nematodes, beetles, aphids, and stinkbugs, can damage crops and devastate yields. Plant pathogenic nematodes alone have caused estimated 100 billion dollars in losses globally each year.^37^ Pest populations can increase rapidly and remain persistent in the fields, affecting crops year after year.^38,39^ Early detection and preventing establishment of nematodes and other insects is important to protect plants and maintain yields.^40^ Poncho® VOTiVO®, sold by the German-based chemical company BASF, is described to protect against nematodes and other insects through a combination of the chemical clothianidin and the microbe *Bacillus firmus*.^25,26,41^ *Bacillus firmus* is non-engineered in Poncho®VOTiVO® and has natural plant beneficial and nematocidal properties.^41^ The product is administered as a seed treatment through which the bacteria compete with the nematodes preventing them from colonizing.^41^ In addition, *Bacillus firmus* colonizes plant roots, forming a barrier that protects the plant and promotes healthy root development.^41^ While Poncho® VOTiVO® does not contain a genetically engineered microbe, a second iteration called Poncho® VOTiVO® 2.0 includes a genetically engineered *Bacillus thuringiensis*.^25^ For our purposes, we have focused on the original Poncho® VOTiVO® product to contrast the difference between a genetically engineered and a non-engineered microbe.

### BiomElixOne® from Folium Science

Salmonellosis is a significant foodborne illness usually contracted by consuming *Salmonella*-contaminated food such as poultry and eggs.^42^ *Salmonella* is often found in the gut of poultry animals.^43^ While antibiotics can be used to kill or inhibit *Salmonella* growth in poultry animals, concerns about antibiotic resistance and the desire of consumers to eat meat without antibiotics have led to a need for other methods to control *Salmonella* outbreaks.^42,43^ BiomElixOne® is a food additive with genetically engineered *Escherichia coli* that contains plasmids with gene sequences that target *Salmonella* and other harmful pathogens in poultry.^44^ BiomElixOne was created by the United Kingdom-based biotechnology company Folium Science and was developed using guided biotics. Using gene editing techniques like CRISPR/Cas, Folium Science created plasmids with Cas arrays that target *Salmonella* and other harmful pathogens to specifically kill or inhibit pathogenic microbes in the guts of poultry animals without harming the beneficial microbes.^44^ The genetically engineered *Escherichia coli* is fed to the animals where the target-specific plasmids can be transferred into other bacteria within the gut, resulting in the activation of targeted recipient bacteria’s own CRISPR system to lethally damage its genomic DNA.^44^ Through this process, the undesired bacteria can be removed or reduced.

Genetically engineered microbes (GEMs) like the *Kosakonia sacchari* and *Klebsiella variicola* in PROVEN® 40 and the *Escherichia coli* in BiomElixOne® can promote crop and food animal health, increasing agricultural production by using fewer chemicals. The development of new and precise genetic engineering techniques, such as the use of CRISPR/Cas for genome editing, have spurred the use of GEMs in agriculture^45^ in a variety of contexts including plant and animal health, bioremediation and soil health, biological controls for pests, pathogens, and weeds,^46^ and as biofertilizers.^47^

Several features of the above technologies are important to note and affect regulatory review. For example, the microbes in PROVEN® 40 do not contain any genetic material that is foreign to the microbe or come from a different genus of microbe. The technology is not a pesticide nor is the microbes considered to be plant pests. Finally, while not intended to be consumed on its own, it is possible for the technology to be consumed by humans or animals. In contrast, the microbes in Poncho® VOTiVO® are not engineered but are combined with clothianidin to be used as a pesticide and seed treatment. In BiomElixOne® the microbes are used as a vector to transfer *Salmonella-*targeting Cas arrays to other microbes found in poultry animal guts. These features of use, organisms, and genes form the core considerations of regulatory review in the U.S. under the Coordinated Framework for the Regulation of Biotechnology.

### The Coordinated Framework for the Regulation of Biotechnology

**Figure 1.**
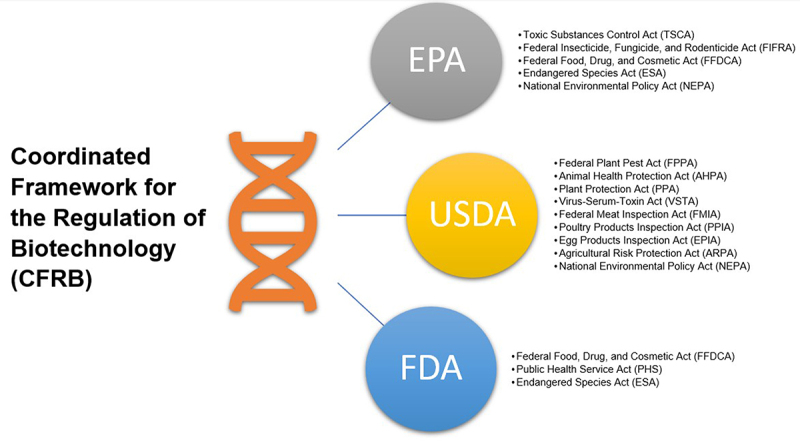
Three administrative agencies, the Environmental Protection Agency (EPA), the United States Department of Agriculture (USDA), and the Food and Drug Administration (FDA), make up the Coordinated Framework for the Regulation of Biotechnology. A variety of legislation provides each agency with its authority for oversight.

The Coordinated Framework for the Regulation of Biotechnology (CFRB) is the U.S. regulatory review system through which biotechnology is approved for public release.^48,49^ Established in 1986 by the Office of Science and Technology Policy, CFRB authorizes three agencies to review and determine the safety of biotechnology. The three agencies are the Environmental Protection Agency (EPA), the United States Department of Agriculture (USDA), and the Food and Drug Administration (FDA).^49–51^ Explicit authority for these agencies to assess technology, create rules and provide guidance on technology development comes from several pieces of legislation, including the Toxic Substances Control Act (TSCA), the Federal Insecticide, Fungicide and Rodenticide Act (FIFRA), the Federal Plant Pest Act (FPPA), the Public Health Service Act, and the Federal Food Drug and Cosmetic Act (FFDCA), among others (Figure 1). Under the CFRB, these agencies focus on the product instead of the process by which the product is made.^49–51^ For example, a technology modified genetically through CRISPR but that contains no new or foreign genetic material is treated differently than a technology that undergoes the same process but contains foreign genetic material.^49–51^

Over time, the CFRB evolved to address emerging technologies, such as gene-edited organisms, but there is still debate and criticism over whether these changes to the CFRB balance competing interests between safety, risk and innovation.^29–31^ Furthermore, new discourse has arisen over whether the CFRB sufficiently considers factors like equity, transparency, and social acceptance.^52–54^ As the development of GEMs increases, it becomes necessary to assess the CFRB, review its strengths and weaknesses, and determine whether the framework can still effectively support evolving technology and evolving interests.

### The CHIPS and Science Act and the 2022 Executive Order on the Bioeconomy

The next evolution of the CFRB is foreshadowed in the Creating Helpful Incentives to Produce Semiconductors (CHIPS) and Science Act and the 2022 Executive Order on the Bioeconomy. On August 9, 2022, President Joe Biden signed into the law the CHIPS and Science Act.^32^ A key section of the act calls for review and changes to bioeconomy research and development.^32^ Specifically, the Act creates a National Engineering Biology Research and Development Initiative that is tasked with providing grants, support, resources and training to promote biological engineering.^32^ In addition, the Act also calls for the review of biological engineering research and development, taking into consideration ethical, legal, environmental, safety, security and social issues.^32^ The law allows for the broadening of regulatory considerations, expanding beyond scientific, technical and economic factors for risk assessment put forth in the 2019 Executive Order on Modernizing the Regulatory Framework for Agricultural Biotechnology Products (2019 Executive Order).^55^ Furthermore, it provides the necessary legislative authority for CFRB agencies to promulgate new rules and guidelines based on ethical and social considerations.^32^

The expansion and authority are elaborated on in the September 12, 2022, Executive Order 14,081 on Advancing Biotechnology and Biomanufacturing Innovation for a Sustainable, Safe and Secure American Bioeconomy (2022 Executive Order).^33^ The 2022 Executive Order recognizes that uses for biotechnology and biomanufacturing should be ethical and responsible while also setting forth policy goals to “clarify and streamline regulations in service of a science- and risk-based, predictable, efficient, and transparent system to support the safe use of products of biotechnology” and “promote standards, establish metrics, and develop systems to grow and assess the state of the bioeconomy; to better inform policy, decision-making, and investments in the bioeconomy; and to ensure equitable and ethical development of the bioeconomy.”^33^ Specifically, the 2022 Executive Order tasks the heads of CFRB agencies to identify “ambiguities, gaps, or uncertainties” in the January 2017 update to CFRB and the 2019 Executive Order to clarify and improve regulatory oversight while also furthering societal goals such as health, climate change and food security.^33^ Furthermore, the agencies are encouraged to conduct this review through stakeholder engagement, which facilitates and increases the likelihood of including diverse criteria and considerations in the regulatory review process.^33^

Recommendations and planned initiatives to reform CFRB as directed under the CHIPS and Science Act and the 2022 Executive Order were submitted by the President’s Council of Advisors on Science and Technology in the December 2022 Report to the President on Biomanufacturing to Advance the Bioeconomy (2022 Report).^56^ The report notes Pivot Bio as an illustrative example of a company that can support agriculture through symbiotic microbial supplements, boosting the bioeconomy.^56^ With respect to CFRB, the 2022 Report notes that regulatory uncertainty is harming U.S. innovation and that new regulatory processes are necessary to review novel or cross-cutting biotechnologies.^56^ The 2022 Report suggests the creation of a Rapid Response Team to vet and provide regulatory approval route guidance to developers of new, cross-cutting biotechnology and to support operations for the Unified Website for Biotechnology Regulation.^56^ In addition, the 2022 Report recommends the creation of a Bioproducts Interagency Working Group that creates “streamlined and model regulatory pathways and act[s] as a vehicle for sharing promising practices across agencies.”^56^ Finally, the 2022 Report recommends the development of a regulatory scientist network associated with biomanufacturing hubs to enable regulatory scientists to keep abreast of novel technologies and to improve coordination among CFRB agencies during the review process, providing certainty and shortening review time.^56^

To further adhere to the mandates of the 2022 Executive Order, the CFRB agencies published the “Report on Stakeholder Outreach Related to Ambiguities, Gaps, Uncertainties in Regulation of Biotechnology Under the Coordinated Framework” (2023 Stakeholder Report) in March 2023^58^ and a plain language guide “The Coordinated Framework for the Regulation of Biotechnology” (2023 CFRB Guide) in November 2023.^57^ The 2023 Stakeholder Report identifies areas of confusion and uncertainty based on input from stakeholders including biotechnology developers, lobbyists, legal representatives, industry groups, non-governmental organizations and government entities.^58^ The report also illuminates strengths and weaknesses of CFRB, which directly affect the attribute category of the Multi Criteria Decision Analysis framework. Specifically, comments and feedback regarding regulatory clarity, coordination and harmonization, reform and revision and resources are highlighted.^58^ The 2023 CFRB guide provides a background of the CFRB and synthesizes the role each framework agency plays in oversight of biotechnology.^57^ The guide provides key questions asked by each agency to determine if oversight is required and lists the specific agency or group within an agency that would be the leading oversight authority.^57^ Furthermore, the guide provides a set of diverse biotechnology examples to illustrate regulatory oversight authority and reach.^57^

### Multi Criteria Decision Analysis Framework

**Figure 2.**
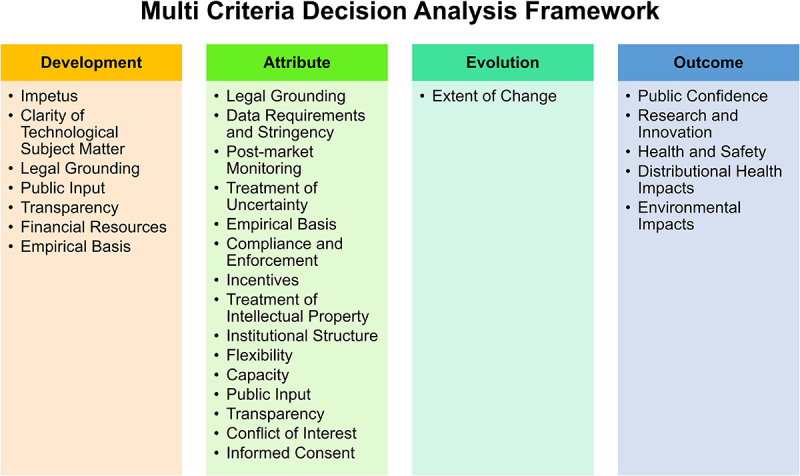
The 28 criteria of the Multi Criteria Decision Analysis (MCDA) Framework from Kuzma et al. 2009 are grouped into four categories and have been used in this commentary to evaluate the current and proposed U.S. regulatory system for biotechnology. Development focuses on how oversight systems are formed. Attribute examines how oversight systems function. Evolution explores how oversight system change over time. Outcome looks at the impacts of the oversight system.

To identify the strengths, weaknesses and effectiveness of the CFRB and its proposed changes with respect to microbial technologies like PROVEN® 40, Poncho® VOTiVO®, and BiomElixOne®, we apply the Multi Criteria Decision Analysis (MCDA) framework. MCDA was developed by Kuzma et al.. (2008, 2009) as part of a U.S. National Science Foundation study to identify important criteria for the evaluation of technology governance and oversight.^30,31^ As applied here, MCDA consists of 28 criteria that have been organized into four categories: development, attribute, evolution and outcome of governance systems (Figure 2).^29–31^ The development examines how the regulatory system was formed. Attribute addresses how the regulatory system functions.^29–31^ Evolution considers how the regulatory system changes over time.^29–31^ Outcome focuses on the impacts of the regulatory system.^29–31^ These criteria were selected and curated using qualitative and quantitative methods, such as direct expert consultation, literature review and stakeholder interviews.^29–31^ The chosen criteria were determined based on those that were rated above 70 on a scale of 1–100 by more than 70% of the experts in the study.^29–31^ Using different types of criteria allows MCDA to not only consider safety, risk and innovation, as CFRB focuses on but also allows for the inclusion of societal impacts and values. As noted by Barry Bozeman and Daniel Sarewitz, the inclusion of societal impacts and values is important for public acceptance of biotechnology.^59^ Without public support, emerging technology adoption can fail just as it would for being risky, unsafe or unprofitable. Furthermore, the inclusion of societal impacts and values help bolster public trust in the regulatory process and system.

### Each microbial technology is subject to different regulatory review. PROVEN® 40 is not subject to regulatory review, while Poncho® VOTiVO® and BiomElixOne® are subject to EPA/USDA and EPA/FDA oversight, respectively.

Each agency of the CFRB has its own pathway, rules and consideration for regulatory review. At this time, there is no centralized pathway through which a technology can be submitted for review by all three agencies concurrently.^49,57^ Consequently, it is necessary to determine whether the technologies are subject to regulatory review under the EPA, USDA, and FDA separately (Figure 1). Agency review can depend on a variety of factors including the organism type, method of genetic engineering, source of genetic material and end use of product.^49,57^

PROVEN® 40 contains living, genetically engineered bacteria with enhanced nitrogen-fixation and colonization capabilities. While *Kosakonia sacchari* and *Klebsiella variicola* are isolated directly from maize roots,^24,36^ they have been genetically altered. These alterations, however, have not introduced new genetic material from other organisms, and the product can best be described as a plant biostimulant as it is intended to help support plant growth and development and provide nutrients. Poncho® VOTiVO® contains living, non-engineered bacteria with natural nematocidal properties.^25,26,41^ No new genetic material is added, but the product is intended as a pesticide.^41^ BiomElixOne® contains genetically engineered bacteria which are fed to poultry animals for human consumption.^44^ In addition, the Cas arrays engineered into the *Escherichia coli* plasmids come from another organism, *Salmonella*.^44^ Altogether, these three products contain differences in their microbes, modifications and uses, resulting in different regulatory outcomes.

### The criteria for EPA review revolves around use of the product and the identity of organisms used for genetic engineering.

The authority of the EPA’s regulation comes from FIFRA, FFDCA and TSCA. FIFRA and FFDCA focus on pesticides in the environment and food, respectively. Here, the threshold question is whether the technology is a pesticide.^57,60^ If it is not, it is not subject to review under FIFRA or FFDCA. Neither PROVEN® 40 nor BiomElixOne® are pesticides as defined by the EPA.^49,57,60^ Consequently, they would not be subject to EPA review under FIFRA. However, BiomElixOne is a feed additive, triggering FFDCA review. In addition, Poncho® VOTiVO® is a pesticide and subject to EPA review. This regulatory trigger, however, is due to the use of the product and not the modification of the microbes involved. TSCA more broadly considers microbial-based products, like PROVEN® 40 and BiomElixOne®. However, the threshold question here is whether the technology contains “new” organisms that is explicitly defined by statute.^49,57,60^ Under the TSCA, a “new” organism is one that may contain synthetic genes from different taxonomic genera.^49,57,60^ This type of organism is known as “intergeneric” and is subject to EPA review.^49,57,60^ However, an organism that has no new genes or genes from the same taxonomic genera is “intrageneric” and not subject to EPA review.^49,57,60^ The microbes in PROVEN® 40 were gene-edited to remove parts of the gene or to enhance gene regulation. No new genetic material was added. Thus, PROVEN® 40 falls under the definition of an “intrageneric” microbial biotechnology and is exempt from EPA review. However, BiomElixOne® is “intrageneric” involving a host from one taxonomic genera and genetic material from another taxonomic genera, thus becoming subject to EPA review. Under TSCA, the focus is on the definition of the organism and its status as a “new” organism without relevance to how the organism may have been altered.^49,57,60^

### The criteria for USDA review revolves around being a plant pest, containing genes from plant pests, or being used to control plant pests.

The key question for USDA regulatory review is whether the genetically engineered microbe is a plant pest or contains plant pest genes or poses a plant pest risk.^49,57,60^ If it does, it is subject to review. The microbes in PROVEN® 40 and BiomElixOne® are not plant pests, do not contain plant pest sequences in their genomes and are not being used to control plant pests.^49,57,60^ Plant pests are explicitly defined as “[a]ny living stage of protozoa, nonhuman animal, parasitic plant, bacterium, fungus, virus or viroid, infectious agent or other pathogen, or any article similar to or allied with any of the foregoing that can directly or indirectly injure, cause damage to, or cause disease in any plant or plant product.”^49,57,60^ If the microbial technology does not meet this definition, it is not considered to be a plant pest. This “not regulated” regulatory status was confirmed by the USDA through a letter received by Pivot Bio that stated it was not subject to oversight due to not posing a plant pest risk.^61^ USDA’s regulatory pathway was also revised in 2020 to focus more on the microbe’s properties and not on the method used to produce it.^49,57,60^ However, Poncho® VOTiVO® is being used to control nematodes and other plant pest insects and is subject to USDA review even though it does not contain a microbe that is a plant pest. Similar to the EPA criteria, the USDA looks to see if the technology falls within its definition for regulatory review.^49,57,60^

### The criteria for FDA review revolves around being an ingredient in human or animal food.

With respect to FDA review, regulatory oversight is a little less clear. A variety of food products are made from microbial fermentation, and the FDA reviews genetically engineered microbes for safety and nutrition, determining whether such foods are “substantially equivalent.”^49,57,60^ PROVEN® 40 and Poncho® VOTiVO® are not food products for humans or animals. However, they are microbial colonizers of maize roots. While there is food derived directly from maize crops, the part of the maize that is typically eaten or used is not the same part where these microbes are present. Furthermore, the plant’s growth and development are improved by the presence and efforts of genetically engineered microbes. If these efforts lead to higher nutritional content or affect the safety of the food, FDA review could be triggered, but as they are now, they are not subject to FDA review. This regulatory status is further confirmed by the 2023 CFRB Guide which uses a technology like PROVEN® 40, an intergeneric nitrogen-fixing soil bacteria, as an example to illustrate CFRB agency review.^49,57,60^ The 2023 CFRB Guide suggests that the only possible regulatory review of an intergeneric nitrogen-fixing soil bacteria comes from the EPA, if the organism is intergeneric, and the USDA, if the organism is a plant pest or has plant pest potential.^49,57,60^ BiomElixOne® is a clear case of FDA review as it is a feed additive and thus an ingredient in animal food.

Due to its characteristics and use, PROVEN® 40 is not subject to regulatory review even though it has two genetically engineered microbes with multiple alterations to their genomes. Poncho® VOTiVO® is subject to EPA and USDA review due to its use as a pesticide and to control a plant pest, respectively. BiomElixOne® is subject to EPA and FDA review due to its characteristic of being an “intrageneric” organism and its use as a feed additive.

### The current U.S. regulatory review process shows weaknesses and areas for improvement, some of which are being directly addressed by the CHIPS and Science Act and the 2022 Executive Order.

While not having to go through the regulatory review process, as in the case of PROVEN® 40, will streamline research, development and distribution of genetically engineered microbial technology, it does raise questions on whether the current system is properly balanced between safety, risk and technological innovation. Furthermore, the criteria for regulatory review solely focus on risk and safety as defined by statute or agency rules.^59^ It does not account for social values that affect acceptance of the technology. To assess the current CFRB and the proposed changes to the CFRB, we apply the MCDA.^29–31^ MCDA was developed as an evaluation tool for regulatory oversight systems.^29–31^ In applying the MCDA, we explored the CFRB and proposed changes by category, providing a numerical value between 1 and 100 (weak to strong) for each criterion in the category. Each score was then categorized into a Strong (61–100), Neither (40–60) or Weak (1–39) designation. The designation of the category was determined by the majority of Strong, Neither or Weak designations of each underlying criterion in that category. Exploring the regulatory pathways under MCDA provides insight into the strengths and weaknesses of CFRB. Ultimately, the ideal regulatory system provides clarity, decreases waiting times for regulatory approval, considers risk and safety, and promotes public acceptance of approved technologies. Use of a tool like MCDA can help ensure that proposed changes to the CFRB meet these goals. To that end, examining the current CFRB and proposed changes can highlight specific areas for reform.

### Under the development category, the CFRB is weak. The proposed changes to the CFRB will strengthen this category, but the extent to which it will do so is unclear.

Under the development category, there are seven criteria: impetus, clarity of technological subject matter, legal grounding, public input, transparency, financial resources and empirical basis.^29–31^ Overall, the development category of CFRB is weak. When exploring the regulatory process for PROVEN® 40, it becomes apparent that the CFRB was not designed to directly address this type of technology. Definitions were created that allowed certain technologies to either be exempt or not subject to regulatory review. This was not the case for Poncho® VOTiVO® and BiomElixOne® where regulatory review was clearer. Furthermore, feedback from stakeholder groups in the 2023 Stakeholder Report^58^ illustrated that there was still uncertainty concerning how to handle genetically engineered microbial technology. When applying the CFRB to PROVEN® 40, it was not clear how the rules and regulations were developed and what perspectives were taken into consideration. While public input was solicited through the comment process, it seems that select public input was incorporated, while input regarding social values was ignored. In addition, the lack of central biotechnology governance laws adds to confusion in the development and implementation of the CFRB. Many different laws, such as the TSCA and FPPA, must be consulted, revised and considered to understand the CFRB as authority and triggers for regulatory review are found in separate laws instead of under one.

While the CFRB makes efforts to define and make clear what technology is subject to review, these efforts are more reactive than proactive. After novel technologies are created, the CFRB makes changes. However, proposed changes to the CFRB like the Rapid Response Team and the Bioproducts Interagency Working Group will allow for a more proactive response to technological innovation.^56^ Recruiting members of these groups from diverse stakeholders as well as functioning under the broader goals of ethics and social impacts will allow for greater public input, transparency and legal grounding in CFRB development. Finally, both the CHIPS and Science Act and the 2022 Executive Order have set aside considerable funding ($174 billion) for science and engineering research (including biotechnology), regulation and training.^32,33^ The proposed changes are likely to make the development category stronger and more comprehensive. Efforts like the 2023 Stakeholder Report also show action toward increasing public input in the development of the CFRB.^58^ Solicitation of stakeholders outside of the comment period indicates that this category will be strengthened by the proposed changes to the CFRB.

### Under the attribute category, the CFRB has gaps. The proposed changes to the CFRB will strengthen this category overall, but there is still work to be done.

Attributes are the largest category of the MCDA with 15 criteria: legal grounding, data requirements and stringency, post-marketing monitoring, treatment of uncertainty, empirical basis, compliance and enforcement, incentives, treatment of intellectual property, institutional structure, flexibility, capacity, public input, transparency, conflict of interest and informed consent.^29–31^ Under the current system, this category is split. Some aspects are strong, such as institutional structure and flexibility, while others are weak such as transparency, conflict of interest and informed consent. The functioning of CFRB is complex, and there is disagreement and confusion even among experts on the appropriate pathway or starting point. Furthermore, the lack of a centralized system means that companies may have to make strategic choices about where to start their regulatory review. This is especially true where technology may also require intellectual property protection. The inefficiencies of the CFRB can result in the completion of a significant portion of the patent term before the technology completes regulatory review. Several gaps can be seen in the attribute category. The lack of a single entry into the regulatory system creates a challenge for those who want to submit a biotechnology for review. The CFRB also does not provide consideration for social values in its assessment, which leads to conflicts of interest. The development of the 2023 plain language guidelines also makes clear that the CFRB was difficult to understand and navigate.^57^ Several weak gaps can be identified such as post-market monitoring, treatment of intellectual property, capacity, public input, transparency, conflict of interest and informed consent.

By consolidating agency representation under the Rapid Response Team, the Bioproducts Interagency Working Group and partnering regulatory scientists with biomanufacturing hubs, the 2022 Executive Order and 2022 Report will strengthen the attributes of CFRB by allowing for greater cross communication between agencies as well as greater opportunities staying up to date on technological innovation.^33,56,58^ Furthermore, the 2023 CFRB Guide also helps to provide clarity and directly addresses public input with respect to uncertainty. Continuing efforts to seek public input, like the 2023 Stakeholder Report, demonstrate a specific action to address gaps. As such, it seems that the proposed changes will strengthen this category in some criteria while leaving other criteria lacking.

### Under the evolution category, the CFRB has room for improvement. The proposed changes appear to address this category in a way to strengthen it.

The evolution category has one criterion: extent of change.^29–31^ CFRB is a dynamic framework, but change has been slow. The explicit mandates to streamline and clarify the CFRB will likely result in a stronger system that responds to technological innovation in a more effective manner. Before 2017, the CFRB was not changed very much since its development in 1986. This more than 30-y time gap illustrates a slow evolution of the CFRB. However, since 2017, changes to the CFRB from individual agencies to congress have been progressing at a much faster pace. Since 2017, there have been executive and legislative action to address the weaknesses and gaps of the CFRB, with a focus primarily on streamlining technological innovation.^55,57,58^

### Under the outcomes category, the CFRB has the most potential for improvement. However, until the proposed changes are implemented, it remains unclear as to the extent this category will be strengthened.

The outcomes category has five criteria: public confidence, research and innovation, health and safety, distributional health impacts and environmental impacts.^29–31^ This category focuses on the impacts of the regulatory system and is perhaps the most visible category. Outcomes are a neutral category where the criteria are neither strong nor weak. While the proposed changes will increase the strength of the other categories, it seems unlikely that much will change in the outcomes category until the changes in the other categories have been made. Many of the proposed changes, especially the interest in incorporating social values comes from considerations in the Outcomes category. The criteria in this category are factors to balance in the regulatory system. The current CFRB only balances risk, safety and technological innovation, but the proposed system seems to add more considerations, which should improve public confidence and environmental impacts. However, until the proposed changes are solidified and implemented, it will be difficult to know to what extent this category could be strengthened.

While it is still too soon to tell, the proposed changes could address many of the weaknesses of the CFRB such as the treatment of uncertainty, research and innovation, data requirements and stringency and impetus. This determination is made based on the directions and recommendations found in the 2022 Executive Order, 2022 Report, the 2023 Stakeholder Report, and the 2023 CFRB Guide.^33,49,57,60^ The proposed changes focus on information gathering about the CFRB to identify areas where the process can be improved or streamlined as well as facilitating cross-agency communication. While the USDA implemented rules in 2020^49^ to make it easier to regulate genetically engineered microbes for agriculture that were not plant pests, the FDA and the EPA have not yet promulgated similar measures to streamline or clarify regulations, though it seems likely that these will be in place.

## Conclusion

PROVEN® 40, Poncho® VOTiVO® and BiomElixOne® are innovative technologies that use microbes to address concerns in agriculture. Yet, each technology is subject to different regulatory review for reasons largely unrelated to the genetic engineering techniques used. Instead, the technologies were subject to regulatory review based on their use and technical definitions of terms (e.g. plant pest, intrageneric) defined in U.S. law. While this may allow for faster movement through the regulatory process, it also hinders public acceptance of technologies and gives the impression that unfamiliar and unknown technologies are introduced without regulatory review. In addition, the need to assess regulatory review under the EPA, USDA, and FDA can create confusion and uncertainty in navigating the regulatory process.^49,57,59^ These weaknesses were further illuminated by the MCDA framework.^29–31^ Many of these weaknesses can be grouped as those related to clarity, transparency, public input, and social acceptance, which suggests important areas for reform. Furthermore, while the current CFRB can accommodate novel technologies like genetically engineered microbes for agricultural biologicals, changes are needed to allow for the accommodation of the next generation of technologies, such as genetically engineered microbiomes. Changes to the CFRB are needed not only to make the regulatory review more effective and efficient but to also facilitate public support and commercialization of technology by taking into consideration ethical and social issues. Improving clarity, decreasing review time, and balancing risk will significantly improve challenges to technology development, but commercialization may still pose an issue without also addressing consumer acceptance and societal concerns. The CHIPS and Science Act and the 2022 Executive Order provide funding, directives, and opportunity to make substantial reform to the CFRB that eliminate uncertainty and bolster trust in the regulatory process while also balancing safety, risk, and innovation.
